# Identifying a combination of biomarkers to predict treatment response to nabilone for the treatment of agitation in Alzheimer's disease – a secondary analysis

**DOI:** 10.1002/alz70857_103580

**Published:** 2025-12-25

**Authors:** Hui Jue (Janet) Wang, Myuri Ruthirakuhan, Nathan Herrmann, Ana C. Andreazza, Damien Gallagher, Nicolaas Paul L.G. Verhoeff, Alex Kiss, Sandra E. Black, Oriel J Feldman, Krista L Lanctôt

**Affiliations:** ^1^ Sunnybrook Research Institute, Toronto, ON, Canada; ^2^ University of Toronto, Toronto, ON, Canada; ^3^ Sunnybrook Health Sciences Centre, Toronto, ON, Canada; ^4^ Center for Addiction and Mental Health, Toronto, ON, Canada; ^5^ Department of Psychiatry, Temerty Faculty of Medicine, University of Toronto, Toronto, ON, Canada; ^6^ Baycrest Hospital, Toronto, ON, Canada

## Abstract

**Background:**

Agitation is a challenging neuropsychiatric symptom (NPS) of Alzheimer's disease (AD). A crossover trial found that nabilone significantly improved agitation in AD patients over 6 weeks compared to placebo. Here, we aim to identify a combination of biomarkers that could be used to predict treatment response to nabilone for AD‐associated agitation.

**Methods:**

Agitation was assessed using the Cohen‐Mansfield Agitation Inventory (CMAI). Serum concentrations of 13 markers were measured. Linear regression was used to estimate change in CMAI due to nabilone for the high and low groups of each biomarker. Biomarkers with a difference ≥8.5 points between groups were included in subsequent multivariate models. Index scores representing the difference between expected CMAI change given nabilone and placebo were calculated and divided into quartiles. Mean difference in CMAI change and 95% confidence intervals were estimated via bootstrapping.

**Results:**

Four of the 13 biomarkers which met criteria specified above were included in multivariate modeling (*n* = 67). Nabilone was more efficacious in participants with higher IL‐6 (estimated change in CMAI ‐15.4, standard error (SE) 5.6), higher ISO‐8 (‐14.4, SE=5.0), higher 24S‐OHC (‐14.2, SE=4.1), and lower clusterin (‐14.6, SE=4.4). Participants in Q1 of index scores demonstrated better response to nabilone with a mean difference in CMAI change of ‐20.9 (95% CI: ‐31.8, ‐9.2), while those in Q2‐4 showed no difference between treatments.

**Conclusions:**

Participants with higher levels of inflammation, oxidative stress, and cholesterol metabolite were more likely to benefit from nabilone for agitation in AD. A combination of biomarkers could help in distinguishing responders and non‐responders to nabilone.

